# Serine ubiquitination of the SARS-CoV-2 RdRp by the non-canonical E3 ligase MYCBP2

**DOI:** 10.1128/msphere.00373-26

**Published:** 2026-09-01

**Authors:** RuthMabel Boytz, Jadon Layne, Cleopatra Skerrit-Genelus, Chengjin Ye, Jack Chun-Chieh Hsu, Luis Martinez-Sobrido, Maudry Laurent-Rolle

**Affiliations:** 1 Department of Microbial Pathogenesis, Yale School of Medicine 5755 , New Haven, New Haven, USA; 2 Lung Biology Institute, Perelman School of Medicine, University of Pennsylvania, Philadelphia, Pennsylvania, USA; 3 Section of Infectious Diseases, Department of Internal Medicine, Yale School of Medicine 5755 , New Haven, Connecticut, USA; 4 Disease Intervention and Prevention, Texas Biomedical Research Institute 7075, San Antonio, Texas, USA; 5 Center for Virus-Host-Innate-Immunity, Institute for Infectious and Inflammatory Diseases, New Jersey Medical School, Rutgers, The State University of New Jersey 12286, Newark, New Jersey, USA; 6 Department of Medicine, New Jersey Medical School, Rutgers New Jersey Medical School, Rutgers, The State University of New Jersey 12286, Newark, New Jersey, USA; Johns Hopkins University Bloomberg School of Public Health, Baltimore, Maryland, USA

**Keywords:** SARS-CoV-2, ubiquitination, coronavirus, RNA-dependent RNA polymerase, posttranslational modifications, MYCBP2

## Abstract

Post-translational modifications, including ubiquitination, have emerged as important regulators of viral infection and the host innate immune response. We screened the proteins of SARS-CoV-2 for ubiquitination and identified the RNA-dependent RNA polymerase (RdRp) NSP12 as being ubiquitinated, which has not previously been reported. Importantly, we confirmed that NSP12 is ubiquitinated during SARS-CoV-2 infection, and the NSP12 proteins of different coronaviruses are differentially associated with ubiquitin chains. SARS-CoV-2 NSP12 was primarily associated with K63-linked polyubiquitin chains, which increased SARS-CoV-2 NSP12 stability relative to OC43 NSP12, which was associated with K48 chains. Additionally, we identify the atypical E3 ubiquitin ligase MYCBP2 as a mediator of serine ubiquitination of SARS-CoV-2 NSP12 on serine-564 and observe that MYCBP2 promotes SARS-CoV-2 replication in cell-based assays. Substitution of this single residue (S564A) in CoV-2 NSP12 prevented recovery of infectious virus in three independent attempts, precluding direct genetic validation of its role in viral replication. To our knowledge, these findings provide the first evidence of serine ubiquitination of a viral protein and suggest a previously unrecognized role for RdRp ubiquitination in coronavirus biology.

**IMPORTANCE:**

Viruses often hijack host post-translational modification systems, including ubiquitination, to enhance the functions of their proteins and evade host innate immune responses. We screened the viral proteins of SARS-CoV-2 and found that NSP2, NSP5, NSP12, NSP15, and NSP16 are ubiquitinated. Focusing on NSP12, the RNA-dependent RNA polymerase (RdRp), we found that it is associated with multiple types of ubiquitin modifications, and RdRp proteins from other coronaviruses are differentially ubiquitinated. We also found that SARS-CoV-2 RdRp undergoes ubiquitination on a serine, rather than a canonical lysine residue, mediated by the atypical E3 ubiquitin ligase, MYCBP2. Consistent with this, MYCBP2 promotes efficient SARS-CoV-2 replication in cell-based assays. To our knowledge, this study provides the first evidence of non-lysine ubiquitination of a viral protein and demonstrates the serine selectivity of MYCBP2 for the first time in a cellular context. These findings raise important questions about the regulatory function of serine NSP12 ubiquitination in coronavirus replication and its potential contribution to viral pathogenicity.

## INTRODUCTION

Viruses encode a limited set of proteins, and post-translational modifications (PTMs) can enhance the functional capabilities of viral proteins. Viruses have evolved sophisticated mechanisms of hijacking the host PTM machinery to enhance their replication. Ubiquitination is essential for innate antiviral immune signaling, and its role in the activation of the RIG-I-like receptors RIG-I and MDA5 and downstream signaling cascades is well-studied (1–6). Unsurprisingly, the ubiquitin system is therefore a frequent target of viral manipulation. These ubiquitin-virus interactions can tip the balance in favor of either the host or the virus and are a key focus of host-virus interaction studies (7–12).

SARS-CoV-2 is no exception. Early studies described SARS-CoV-2-induced alterations to the host ubiquitin landscape (13, 14). More recent studies have focused on understanding the functional roles of ubiquitin and ubiquitin-like modifications of SARS-CoV-2 viral proteins (15–27). The importance of ubiquitination for SARS-CoV-2 infection and the potential translational aspects of this understanding are evidenced by several reviews on the topic (28, 29).

We examined ubiquitination of SARS-CoV-2 non-structural protein 12 (NSP12), the virus’s essential RNA-dependent RNA polymerase (RdRp). Unlike reports on ubiquitination or sumoylation of non-essential viral accessory proteins (30–33), RdRp in other RNA viruses such as influenza A virus (IAV) (34–36) has been shown to be ubiquitinated by host factors and required for efficient activity. Thus, we asked if and how NSP12 ubiquitination influences SARS-CoV-2 replication.

## RESULTS

**Fig 1:**
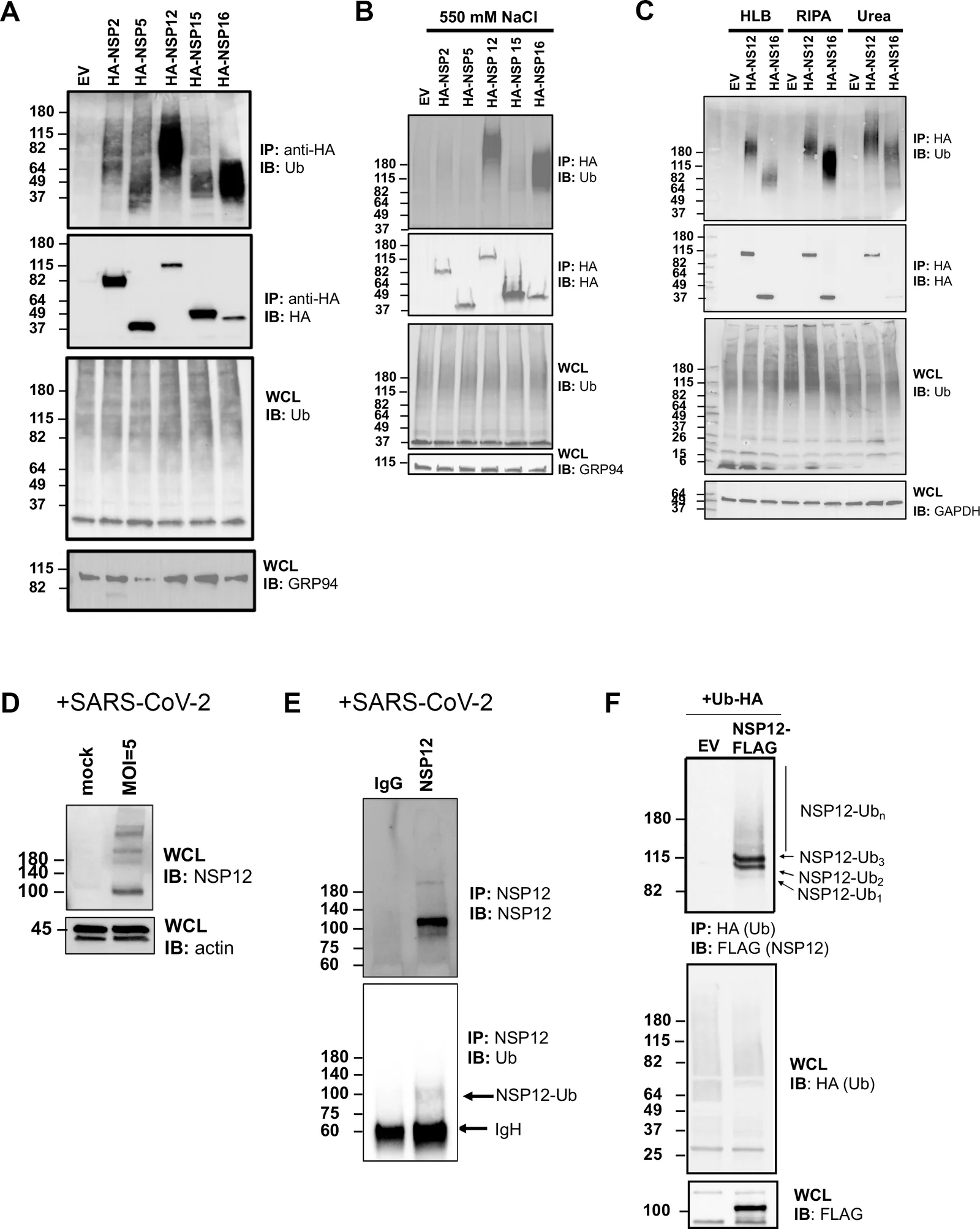
NSP12 is ubiquitinated during SARS-CoV-2 infection. (**A**) HEK293T cells were transfected with HA-tagged SARS-CoV-2 protein expression constructs, followed by anti-HA immunoprecipitation and immunoblotting for ubiquitination. (**B**) The ubiquitinated SARS-CoV-2 proteins identified in Fig. S1A were expressed in HEK293T cells and immunoprecipitated using anti-HA beads under different salt concentrations. Horvath lysis buffer (HLB) contains 280 mM NaCl. A more stringent buffer containing 550 mM NaCl is expected to disrupt non-covalent interactions with the immunoprecipitated protein. (**C**) Proteins were expressed as in panel A under increasingly harsh conditions: HLB, RIPA, or a denaturing 6 M urea buffer (50 mM Tris-HCl pH 8.0 [Sigma], 6 M urea, 350 mM NaCl, 0.5% [vol/vol] IGEPAL CA-630 [Sigma], and 40 mM imidazole), which is expected to disrupt all non-covalent interactions with the immunoprecipitated protein. (**D and E**) VeroE6 cells were infected with SARS-CoV-2 at MOI = 5 for 24 h, followed by immunoprecipitation using an anti-NSP12 antibody or rabbit IgG control. IP and whole-cell lysates (WCLs) were analyzed for ubiquitination by immunoblotting. (**F**) Reciprocal pull-down of HA-tagged ubiquitin followed by immunoblotting to probe for NSP12 association. HEK293T cells were transfected with HA-tagged ubiquitin and FLAG-tagged NSP12.

We initially performed a screen of 20 SARS-CoV-2 proteins. Expression plasmids for each HA-tagged viral protein were transfected into HEK293T cells, and the viral proteins were subjected to immunoprecipitation and western blotting to detect endogenous ubiquitin association (Fig. S1). From this screen, we identified SARS-CoV-2 NSP2, NSP5, NSP12, NSP15, and NSP16 as ubiquitinated when overexpressed in HEK293T cells (Fig. 1A). SARS-CoV-2 NSP5 and NSP16 ubiquitination has previously been described (25, 26, 37). Thus, we focused on SARS-CoV-2 NSP12, as it has not previously been reported to undergo ubiquitination. To rule out the possibility of the NSP12 ubiquitin signal coming from co-precipitating ubiquitinated proteins, and not from NSP12 itself, we immunoprecipitated HA-NSP12 from HEK293T cells using lysis and wash buffers containing different amounts of NP-40 and SDS, high salt concentration, or urea. NSP12 ubiquitination was maintained across all conditions, supporting the conclusion that NSP12 is associated with covalent polyubiquitin chains (Fig. 1B and C). We also performed a double immunoprecipitation experiment, which further confirmed that NSP12 itself is ubiquitinated, and the ubiquitinated species is a minority of the total NSP12 (Fig. S2A and B). We confirmed NSP12 ubiquitination during SARS-CoV-2 infection by immunoprecipitating NSP12 from infected VeroE6 cells (Fig. 1D and E). Two ~100 kDa species were detected, with the slower migrating band corresponding to ubiquitinated NSP12, as confirmed by ubiquitin immunoblotting (Fig. 1E). Reciprocal ubiquitin pull-down revealed multiple higher-molecular-weight NSP12 species and a polyubiquitin-association smear (Fig. 1F). Because endogenous NSP12 was analyzed under conditions optimized for protein recovery (multiplicity of infection [MOI] = 5, 24 h post-infection), the present study does not define the temporal kinetics of NSP12 polyubiquitination during infection. Future studies examining earlier stages of infection will be required to determine whether this modification occurs during the period of active viral RNA synthesis and contributes directly to SARS-CoV-2 replication. Together, these data demonstrate that SARS-CoV-2 NSP12 is ubiquitinated in the context of viral infection.

**Fig 2:**
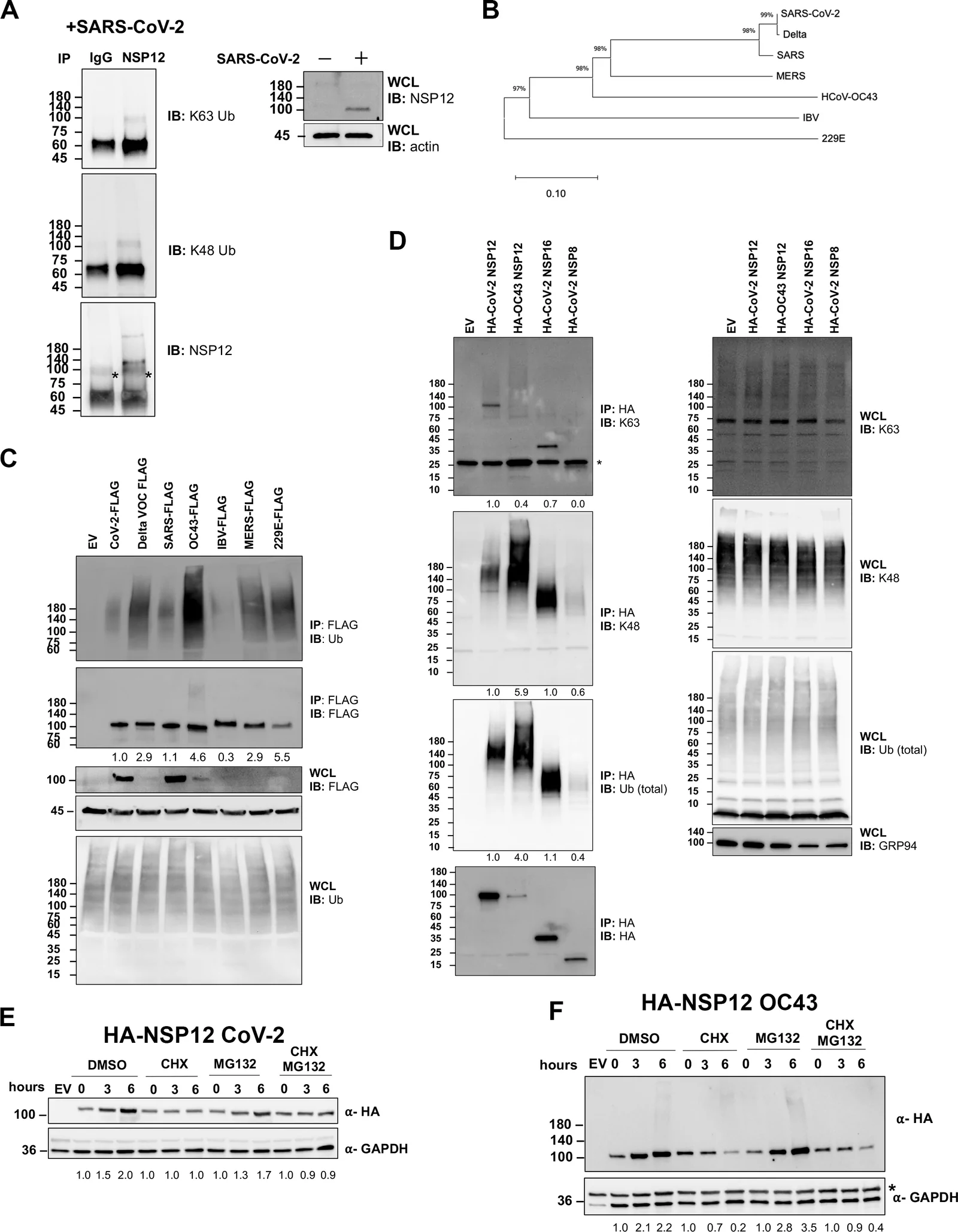
The NSP12 proteins of diverse CoVs are differentially ubiquitinated. (**A**) VeroE6 cells were infected at MOI = 1 for 24 h, followed by immunoprecipitation using an anti-NSP12 antibody. The IP was probed for specific ubiquitin linkages, and the whole-cell lysate (WCL) probed for total NSP12 and actin. Asterisk indicates non-specific band. (**B**) Phylogenetic tree showing evolutionary relationships of the coronavirus NSP12s used in the panel. Scale bar represents evolutionary distance in amino acid substitutions per site. Node values indicate the percentage of 100 bootstraps having the indicated phylogenetic relationship. (**C**) HEK293T cells were transfected with the indicated FLAG-tagged coronavirus NSP12 construct, followed by immunoprecipitation using anti-FLAG beads. The IP and WCL were probed for ubiquitination by immunoblotting. Values represent the mean densitometric intensities of Ub-bands normalized to IP-FLAG bands. (**D**) HEK293T cells were transfected with the indicated HA-tagged expression constructs, followed by IP using anti-HA beads and immunoblotting for specific ubiquitin linkages with linkage-specific antibodies. (**E and F**) NSP12 stability assays. HEK293T cells were transfected with HA-tagged CoV-2 NSP12 (**D**) or OC43 NSP12 (**E**) for 18 h, followed by treatment with the indicated inhibitors (CHX: cycloheximide 50 µg/mL; MG132: 20 mM), and whole cell lysates were collected every 3 h. NSP12 levels in WCLs were analyzed by immunoblotting WCLs. Values represent the mean densitometric intensities of HA bands normalized to GAPDH. An asterisk indicates non-specific binding.

Because polyubiquitin chain topology can dictate substrate fate beyond proteasomal degradation, we next characterized the ubiquitin linkages associated with SARS-CoV-2 NSP12. K48- and K63-linked ubiquitin species were detected on NSP12 immunoprecipitated from SARS-CoV-2-infected cells (Fig. 2A), consistent with the diverse signaling functions attributed to distinct ubiquitin chain types. K48-linked chains are classically associated with proteasomal turnover, whereas K63-linked chains frequently regulate protein trafficking, signaling, and protein-protein interactions.

We next assessed whether NSP12 ubiquitination was conserved across coronaviruses using FLAG-tagged NSP12 constructs from a panel of coronaviruses (Fig. 2B). Ubiquitination levels varied among NSP12 proteins, with notably higher ubiquitination of HCoV-OC43 NSP12 and increased ubiquitination of SARS-CoV-2 Delta variant NSP12 compared to the ancestral SARS-CoV-2 WA1 strain (Fig. 2C). We note that the levels of NSP12-FLAG proteins in the whole-cell lysate (WCL) were not uniform, which is likely due to a combination of different transfection efficiencies and differences in protein stability when overexpressed in HEK293T cells without the addition of cofactors such as NSP7/8. In the case of IBV, reduced stability could be due to the species mismatch between human HEK293T cells and the natural avian hosts for this virus. Despite these different expression levels in the WCL, we successfully loaded equivalent amounts of immunoprecipitated protein (slightly less for IBV), which allowed for comparison of ubiquitination levels across the different coronavirus RdRps.

To compare ubiquitination profiles among coronavirus polymerases, HA-tagged SARS-CoV-2 and OC43 NSP12 proteins were expressed in HEK293T cells, immunoprecipitated, and analyzed using linkage-specific ubiquitin antibodies. SARS-CoV-2 NSP8, previously shown to undergo K48-linked ubiquitination, served as a positive control (15). Both SARS-CoV-2 and OC43 NSP12 were associated with K48-linked ubiquitin chains; however, K48-linked ubiquitination was more prominent on OC43 NSP12. In contrast, SARS-CoV-2 NSP12 exhibited a stronger association with K63-linked ubiquitin chains than OC43 NSP12 (Fig. 2D). These findings indicate that although both polymerases undergo K48- and K63-linked ubiquitination, the relative abundance of these linkage types differs between SARS-CoV-2 and OC43 NSP12.

Given their differential K48 association, we tested whether the stability differed between SARS-CoV-2 and OC43 NSP12. HEK293T cells expressing SARS-CoV-2 or OC43 HA-NSP12 were treated with proteasomal and/or translation inhibitors, and NSP12 levels were monitored over 6 h. SARS-CoV-2 NSP12 was stable following cycloheximide treatment, whereas OC43 NSP12 was rapidly degraded and stabilized by MG132 treatment (Fig. 2E and F). Thus, OC43 NSP12 exhibits reduced stability compared to SARS-CoV-2 NSP12 in this system, consistent with its increased association with K48-linked ubiquitin chains.

**Fig 3:**
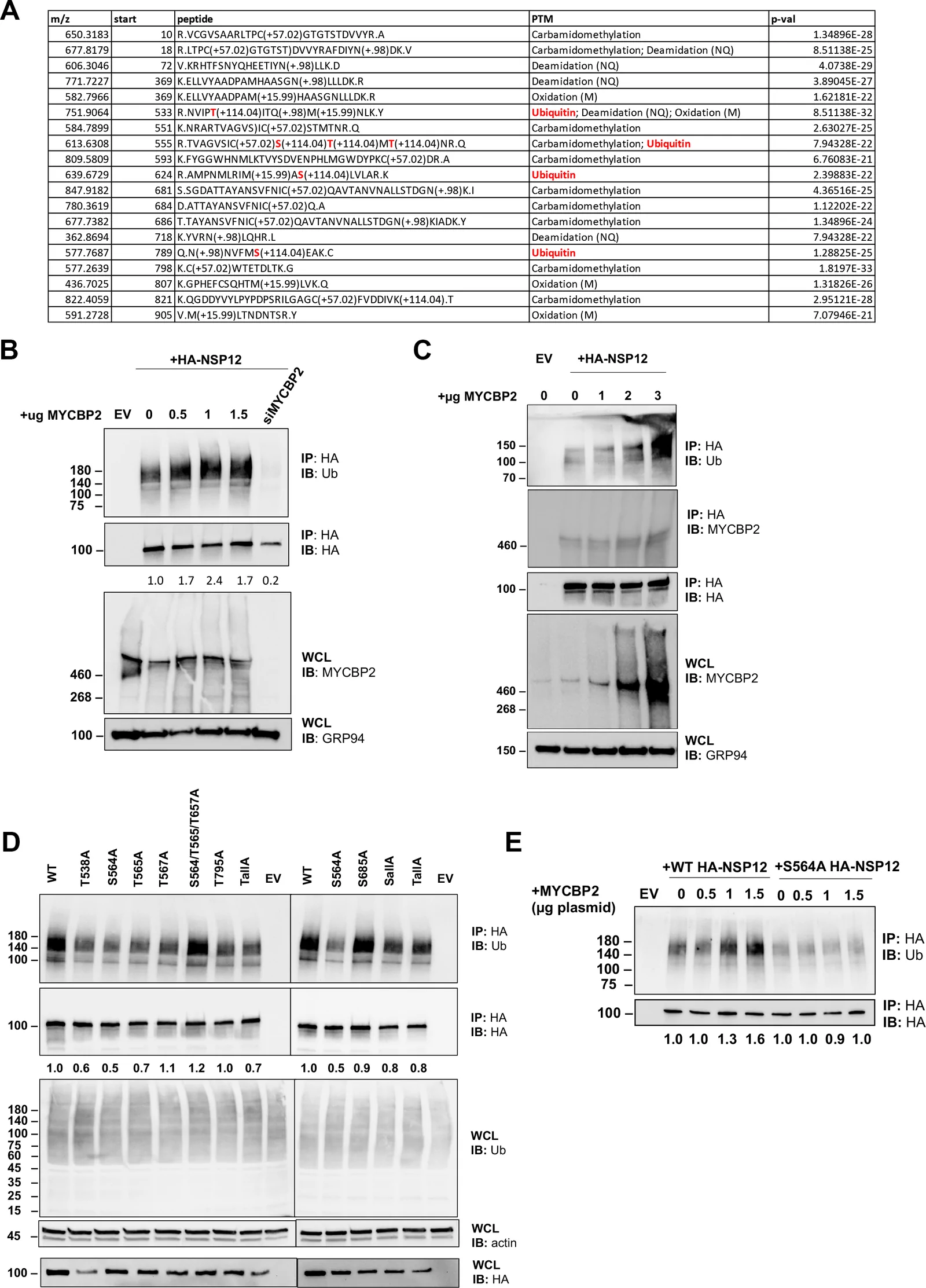
MYCBP2 ubiquitinates NSP12 at S564. (**A**) Post-translational modifications associated with NSP12 identified by tandem LC-MS/MS. NSP12 was immunoprecipitated from infected VeroE6 cells, resolved by SDS-PAGE, and analyzed by LC-MS/MS. Data were analyzed in PEAKS DB to calculate *P*-values. (**B**) HEK293T cells were transfected with increasing amounts of MYCBP2 plasmid and a constant amount of HA-NSP12, or MYCBP2 was knocked down prior to and during NSP12 transfection. NSP12 was immunoprecipitated using anti-HA beads, and the IPs were probed for ubiquitination. Values represent the mean densitometric intensities of Ub bands normalized to HA bands. (**C**) HEK293T cells were transfected with HA-NSP12 and increasing amounts of MYCBP2 plasmid, followed by immunoprecipitation using anti-HA beads. The IPs were probed for MYCBP2 co-precipitation and ubiquitination by immunoblotting. (**D**) Biochemical confirmation of ubiquitinated sites identified by LC-MS/MS analysis, peptides shown in panel A. HA-tagged expression constructs encoding the mutant NSP12s were transfected into HEK293T cells, followed by anti-HA IP and immunoblotting for ubiquitination. Values represent the mean densitometric intensities of Ub-bands normalized to HA bands. (**E**) HEK293T cells were transfected with HA-WT-NSP12 or HA-S564A-NSP12 with increasing amounts of MYCBP2 plasmid, followed by anti-HA immunoprecipitation. IPs were analyzed for ubiquitination by immunoblotting.

Ubiquitination requires a cascade of three enzymes: an E1 activating enzyme, an E2 conjugating enzyme, and an E3 ligase. The E3 ligase imparts substrate selectivity to the process and, together with their cognate E2s, determines ubiquitin linkage type and functional outcomes. We therefore sought to identify the E3 ligase(s) responsible for SARS-CoV-2 NSP12 ubiquitination. Leveraging the SARS-CoV-2 protein interactome published by Gordon et al. (38), we identified MYCBP2, a ~514 kDa atypical RING-Cys-relay (RCR) E3 ubiquitin ligase that catalyzes ubiquitin transfer to serine and threonine residues through a unique relay mechanism, as a candidate E3 ligase for NSP12 (39, 40). MYCBP2 was the only E3 ligase reported to interact with NSP12. This was consistent with our mass-spectrometry data identifying ubiquitinated serine and threonine residues on NSP12 during SARS-CoV-2 infection (Fig. 3A).

MYCBP2 knockdown markedly reduced SARS-CoV-2 NSP12 ubiquitination in transfected HEK293T cells, whereas MYCBP2 overexpression increased SARS-CoV-2 NSP12 ubiquitination in a dose-dependent manner (Fig. 3B and C). Mass spectrometry identified several ubiquitination sites in SARS-CoV-2 NSP12 (Fig. 3A). To confirm that these sites are ubiquitinated, we generated point mutations to an HA-NSP12 construct and probed for the effects on total ubiquitin levels. Multiple individual point mutations reduced NSP12 ubiquitination, with S564A having the largest effect (Fig. 3D). We therefore chose to follow up on the significance of this residue. Consistently, MYCBP2 overexpression did not enhance ubiquitination of an NSP12 S564A mutant, suggesting that MYCBP2 targets NSP12 at residue S564 (Fig. 3D and E). While individual point mutations to NSP12 reduced the level of ubiquitination, a triple mutant (S564A/T565A/T567A) restored ubiquitin levels. We hypothesize that this is a result of the activation of cryptic or compensatory ubiquitination sites, a commonly observed phenomenon in the field in which the mutation of preferred ubiquitination sites results in the targeting of new residues for ubiquitination due to structural changes in the target protein (18, 35, 41, 42).

**Fig 4:**
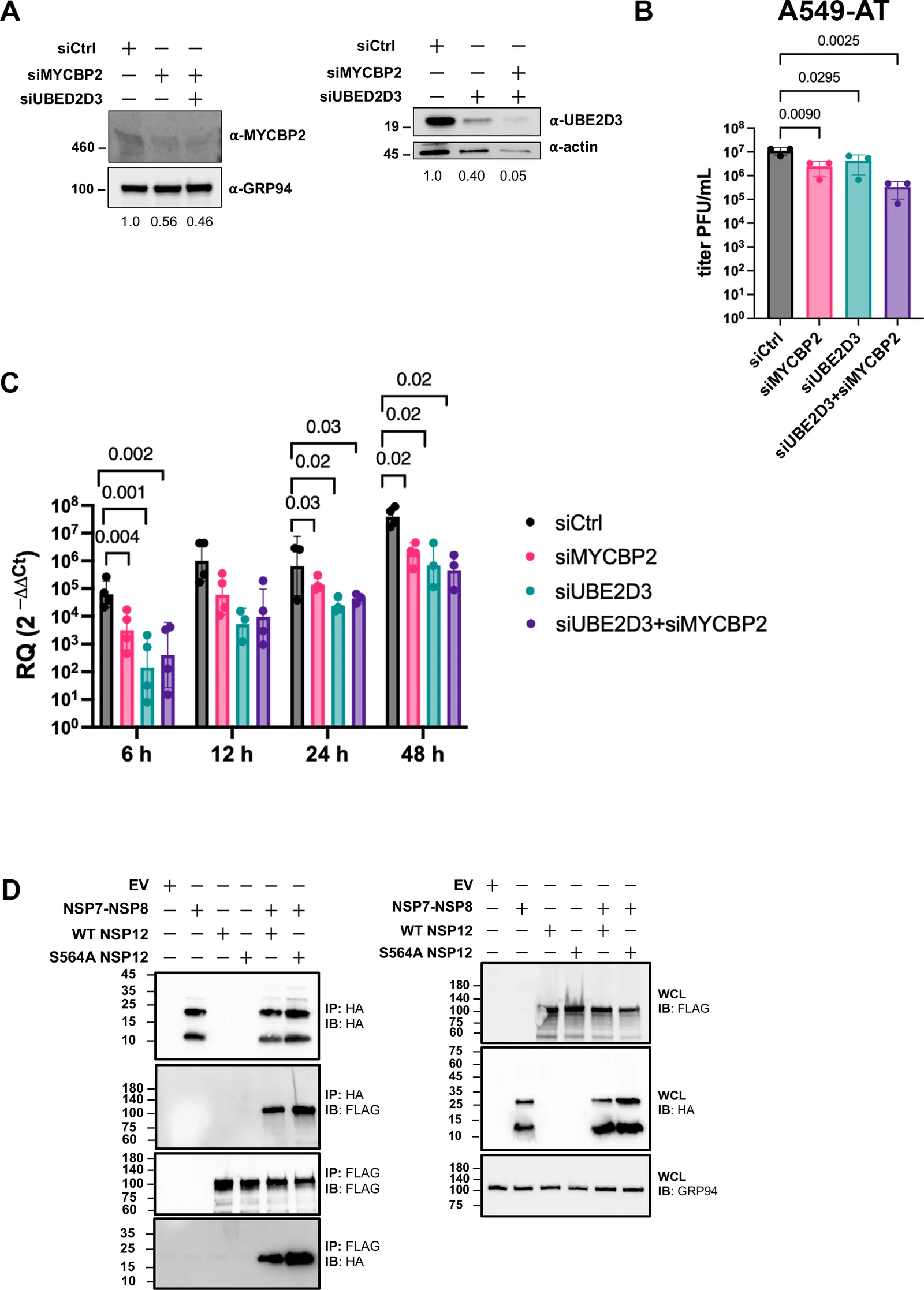
The UBE2D3-MYCBP2 axis is utilized for efficient SARS-CoV-2 replication. (**A**) Confirmation of siRNA-mediated knockdown in A549-AT cells. Densitometry analysis of protein of interest normalized to loading control. (**B**) A549-AT cells were treated with the indicated siRNA for 48 h prior to infection with SARS-CoV-2 at MOI = 0.1 for 48 h. Supernatants were collected and analyzed by plaque assay to measure viral titers. Statistical significance was determined by Dunnett’s multiple comparison test. (**C**) Viral genome kinetics measured by qRT-PCR. A549-AT cells were treated with siRNA and infected as described in panel B. Statistical significance was determined by Dunnett’s multiple comparison test. (**D**) HEK293T cells were transfected with FLAG-WT-NSP12 or FLAG-S564A-NSP12, with or without co-transfection with HA-tagged NSP7 and NSP8, followed by anti-HA or anti-FLAG immunoprecipitation. Interaction between NSP7-8 and WT or mutant NSP12 was analyzed by immunoblotting.

To assess the contribution of MYCBP2-mediated ubiquitination to SARS-CoV-2 replication, we initially attempted to generate MYCBP2 knockout cell lines; however, these displayed a severe growth defect. We therefore performed siRNA-mediated knockdown in A549-AT cells (Fig. 4A). Knockdown of MYCBP2 reduced viral titers by ~0.7–1 log at 48 h post-infection, while knockdown of the E2 conjugating enzyme UBE2D3 reduced SARS-CoV-2 titers by ~0.4 log compared with a scramble control (Fig. 4B). Notably, despite achieving only 46% knockdown of MYCBP2 expression, potentially reflecting the large size and stability of the protein, we observed a statistically significant reduction in viral replication in siMYCBP2 cells. These findings suggest that more complete depletion of MYCBP2 would be expected to produce an even greater replication defect. Combined knockdown of UBE2D3 and MYCBP2 resulted in an ~1.5 log reduction in viral titers, which could suggest that the UBE2D3-MYCBP2 axis contributes to efficient SARS-CoV-2 replication (Fig. 4B). However, as the double knockdown reduced cell viability, the effect on viral replication could be due to impaired cell growth. As MYCBP2 knockdown did not result in cell loss, we can conclude that MYCBP2 contributes to efficient SARS-CoV-2 replication. Given the role of NSP12 in viral genome replication and transcription, we hypothesized that knockdown of MYCBP2 might affect NSP12 activity at early timepoints in infection. To measure this, we performed qRT-PCR on cDNA synthesized from RNA samples collected from si-treated cells at 0, 6, 12, 24, and 48 h post-infection. In support of our hypothesis, the most significant differences in viral genome levels between MYCBP2 knockdown cells and mock-treated cells were observed at 6 h post-infection (Fig. 4C). Consistent with our plaque assay results, knockdown of MYCBP2 alone reduced viral genome levels at 48 h post-infection, with double knockdown of UBE2D3 and MYCBP2 having a larger effect (Fig. 4C), although the reduction in cell viability may confound this effect. Although the differences in viral genome levels at 12 h post-infection did not reach statistical significance due to larger variability in the replicates, we observed the same trend as at 6, 24, and 48 h.

Attempts to rescue a recombinant SARS-CoV-2 expressing NSP12 S564A using previously described and validated bacterial artificial chromosome-based reverse genetics (43) were unsuccessful in three independent attempts, whereas wild-type SARS-CoV-2 was consistently recovered under the same conditions, preventing direct assessment of the role of S564 ubiquitination in viral replication. The inability to recover the mutant virus is unlikely due to impaired replication-transcription complex assembly, as mutant S564A NSP12 retained interaction with NSP8 (Fig. 4D).

## DISCUSSION

In this study, we found that SARS-CoV-2 NSP12 is ubiquitinated during infection and modified by both K48- and K63-linked polyubiquitin chains, whereas OC43 NSP12 is predominantly modified by K48-linked chains, consistent with reduced stability relative to SARS-CoV-2 NSP12. We further identified SARS-CoV-2 NSP12 S564 as a site of serine ubiquitination mediated by the E3 ligase MYCBP2 and observed that this modification is associated with enhanced SARS-CoV-2 replication in cell-based assays. To our knowledge, this represents the first report of serine ubiquitination on a viral protein. Additionally, although MYCBP2 has previously been characterized biochemically for serine/threonine ubiquitin transfer activity in cell-free systems, our findings provide evidence that MYCBP2 targets serine/threonine residues in a cellular context.

Previous studies reported SARS-CoV-2 NSP12 ubiquitination following heat and arsenic trioxide treatment (44, 45). Under those conditions, Maimaitiyiming et al. and Yang et al. found that ubiquitination promoted NSP12 degradation and reduced viral titers. In contrast, in the context of SARS-CoV-2 infection and without destabilizing stimuli, we found that NSP12 is stable against proteasomal degradation, and ubiquitination appears to promote viral replication.

Based on NSP12-NSP7-NSP8-RNA structures, we hypothesize that S564 ubiquitination in SARS-CoV-2 NSP12 may regulate RNA binding, as reported for IAV polymerase (36). The site is located directly adjacent to residues that interact with RNA in the binding groove (Fig. S3B). Attempts to rescue a recombinant SARS-CoV-2 with the S564A mutation in NSP12 were unsuccessful in three different attempts, despite consistent recovery of wild-type virus under identical conditions. While this may suggest an important functional constraint at this position, as the residue is highly conserved across coronaviruses (Fig. S3A), it cannot be genetically confirmed in the context of infectious virus. The inability to recover the mutant virus is unlikely due to major structural destabilization, as S564 resides within an ⍺-helix, and alanine substitutions stabilize ⍺-helices. Although S564 is not on the protein surface in NSP12’s folded equilibrium conformation, it may still potentially be accessible for ubiquitination during folding prior to NSP12 achieving its final conformation. We also note that additional residues are likely ubiquitinated on NSP12 as indicated by the mass spectrometry and western blotting results of NSP12 mutants. Although our findings suggest that S564 is a major contributor to NSP12 ubiquitination, future studies will be needed to determine the relative contributions of additional ubiquitination sites to NSP12 modification and viral replication. One unanswered question raised by our results is where MYCBP2 and NSP12 interact within the cell. Viral RNA replication and transcription occur in convoluted membranes remodeled from the ER, and NSP12 is primarily sequestered in these viral double-membrane vesicles (DMVs) (46–48). MYCBP2 is primarily cytoplasmic with some nuclear localization under certain signaling conditions (49, 50). Given the large size of MYCBP2, it is unlikely to be able to enter DMVs, and furthermore, there is no evidence that this occurs. We therefore hypothesize that MYCBP2 ubiquitinates NSP12 after NSP12 translation in the cytoplasm but before NSP12 is recruited to DMVs. Future studies using high-resolution microscopy and sub-cellular fractionation would improve our understanding of when NSP12 ubiquitination occurs during the viral life cycle and what function this serves for the host and virus.

Ubiquitin regulation of viral RdRp activity has been reported for other viruses. The polymerase subunits of IAV are ubiquitinated by K48, K63, and K29 linkages, and this regulates dynamic structural changes, viral RNA and NP binding, and polymerase dimerization, all of which promote viral replication and gene expression (34–36). Our results demonstrated that the ubiquitination of SARS-CoV-2 NSP12 does not serve a primarily degradative function. Similarly, the polymerase of Senecavirus A is ubiquitinated, and this stabilizes and prevents its proteasomal degradation (51). Knockout of the host E3 ligase responsible for polymerase ubiquitination reduces Senecavirus A viral titers, similar to what we observed with MYCBP2 silencing with SARS-CoV-2 infection.

Although our study establishes a role for MYCBP2-mediated ubiquitination of NSP12 in regulating coronavirus replication, several important questions remain. The molecular consequences of S564 ubiquitination on NSP12 activity have not yet been defined and should be addressed through future analyses of RNA binding, polymerase function, protein conformation, and replicase complex assembly. In addition, our observations suggest that differential NSP12 ubiquitination may contribute to phenotypic differences among coronaviruses and SARS-CoV-2 variants. Determining whether variation in NSP12 ubiquitination influences viral fitness, replication dynamics, or pathogenicity represents an important direction for future studies.

## MATERIALS AND METHODS

### Cell culture

All cells were maintained in Dulbecco’s modified Eagle medium (DMEM) (Gibco) supplemented with 10% fetal bovine serum (FBS) at 37°C, 5% CO_2_. Viral infections were performed in DMEM supplemented with 2% FBS.

### Plasmids

Coronavirus NSP12 sequences were obtained from GenBank, synthesized by Twist Biosciences, and cloned into a pCMV vector with either an N-terminal HA tag or a C-terminal 3×FLAG tag. The MYCBP2 plasmid was obtained from Dr. C. James Hastie of the MRC PPU at the University of Dundee.

### Transfections and immunoprecipitations

HEK293T cells were transfected with Lipofectamine 2000 (Thermo Fisher) for 24 h. Cells were lysed in a lysis buffer containing 0.5% NP40. Cleared lysates were incubated with EZview Red anti-HA or FLAG beads (Millipore; see Table 2), or primary antibody, for 2–24 h at 4°C with end-over-end rotation. After incubation with primary antibody, lysates were incubated with protein A/G agarose beads for 2 h. Beads were washed with lysis buffer, except for MYCBP2 experiments, for which a 0.05% NP40 buffer was used, and resuspended in 2× Laemmli buffer (BioRad).

### Western blotting

MYCBP2 samples were run on NuPAGE tris-acetate mini protein gels, 3%–8% gradient, using NuPAGE running buffer (Thermo Fisher). Proteins were transferred to polyvinylidene difluoride (PVDF) membranes using 20 V for 16 h using NuPAGE transfer buffer. For all other experiments, tris-glycine Mini-PROTEAN gels (BioRad) were used, and transfers were performed using the BioRad Trans-Blot Turbo transfer system. Membranes were blocked with TBST containing 5% bovine serum albumin (BSA) for 1 h at room temperature, incubated with primary antibodies overnight at 4°C (see Table 1 for antibodies and dilutions). After 3× washes, membranes were incubated with horseradish peroxidase (HRP)-conjugated secondary antibodies for 1 h at room temperature, developed with SuperSignal West HRP (Thermo Fisher) substrate, and imaged using a Chemidoc MP. Molecular weight markers were carefully verified for each blot.

**TABLE 1:** Key resource table—antibodies

Antibody	Source	Cat no.	Dilution
Rabbit mAb anti-ubiquitin	Cell Signaling	43124	1:1,000
Rabbit pAb anti-UBE2D1/2/3	Proteintech	11677-1-AP	1:500
Goat anti-rat IgG HRP	Jackson Immunoresearch	112-035-167	1:1,000
Goat anti-rabbit IgG HRP	Jackson Immunoresearch	111-035-144	1:5,000
Goat anti-mouse IgG HRP	Jackson Immunoresearch	115-035-146	1:5,000
Rabbit mAb anti-K63 polyubiquitin	Cell Signaling	5621	1:500
Rabbit mAb anti-K48 polyubiquitin	Cell Signaling	8081	1:500
Rabbit pAb anti-MYCBP2	Abcam	ab86078	1:1,000
Rat mAb anti-GRP94	Enzo	ADI-SPA-850-D	1:1,000
Rabbit pAb anti-NSP12	GeneTex	GTX135467	1:1,000 (WB); 1:50 (IP)
Normal rabbit IgG	Cell Signaling	2729	1:50 (IP)
Rabbit pAb anti-HA	Millipore Sigma	Sigma Aldrich	1:1,000
Rabbit pAb anti-FLAG	Millipore Sigma	F7425	1:1,000
Mouse mAb anti-beta actin	Proteintech	66009-1-Ig	1:5,000

### Viral protein ubiquitination screen

A SARS-CoV-2 plasmid expression library was kindly obtained from Dr. Adolfo Garcia-Sastre (Mt. Sinai). HEK293T cells were transfected with each individual HA-tagged protein as described above, and immunoprecipitations were performed using anti-HA beads as described. Lysates and pull-downs were subjected to western blotting as described above and probed with an anti-HA or anti-Ub antibody to identify ubiquitinated proteins.

### Sequential immunoprecipitation

Total FLAG-NSP12 was immunoprecipitated from HEK293T cells as described in “Transfections and immunoprecipitations.” The beads were pelleted by centrifugation, then incubated with 1% SDS containing 5 mM dithiothreitol (DTT) at 100°C for 5 min. The DTT was inactivated with 1% Triton X-100 in 0.15 M NaCl, 25 mM Tris, pH 7.4. Beads were pelleted, and the supernatant was removed, transferred to a new tube containing the anti-HA beads, and the mixture was incubated for 60 min at 4°C with end-over-end rotation. After three washes in 0.1% Triton X-100 in TBS, the samples were heated in 2× Laemmli buffer and analyzed by western blotting.

### Multiple sequence alignment and phylogenetic tree

Coronavirus NSP12 sequences (AMO03400; AFR58671.1; ACJ12832.1; ATG21344.1; QHU79203.1; AAT8435.1) were downloaded from GenBank. A multiple sequence was generated using Clustal Omega version 1.2.4. The alignment was used to construct a neighbor-joining tree in MegaX using the following parameters: 100 bootstraps, Poisson distribution, and gamma distance rate of 1.

### Mass spectrometry

VeroE6 cells were infected with SARS-CoV-2 WA1 strain at MOI = 5 for 24 h. Cell lysates were immunoprecipitated with an anti-NSP12 antibody (GeneTex). Lysates were subjected to SDS-PAGE, stained with colloidal Coomassie, and bands were excised and sent to the Whitehead Institute at MIT for analysis. Data were analyzed in PEAKS DB.

### Plaque assays

Plaque assays were performed on VeroE6 cells using an overlay medium containing DMEM, 2% FBS, 0.6% Avicel (see Table 2). Plaques were read using crystal violet staining after 48 h post-infection and titers calculated accordingly.

**TABLE 2:** Key resource table—reagents

Reagent	Source	Cat no.
Ezview Red Anti-HA Affinity Gel	Millipore Sigma	E6779
Lipofectamine 2000	Thermo Fisher	11668019
Lipofectamine 3000	Thermo Fisher	L300008
Lipofectamine RNAiMAX	Thermo Fisher	13778075
Ube2D3 Silencer Select siRNA assay	Thermo Fisher	s14579
MYCBP2 Silencer Select siRNA assay	Thermo Fisher	s22982
Silencer Select Negative Control siRNA	Thermo Fisher	4390843
Protease Inhibitor Cocktail complete EDTA-free	Roche	11873580001
4× Laemmli sample buffer	BioRad	1610747
TBS-T 10×	Apex Bioresearch	18-235B
5× Trans-Blot Turbo Transfer Buffer	BioRad	10026938
ImProm-II Reverse Transcriptase	Promega	A3801
MgCl_2_ 25 mM	Promega	A3511
AmpliTaq Gold DNA polymerase	Thermo Fisher	4311806
Random hexamer primer mix	Roche	11034731001
DMEM powder high glucose	Gibco	12100046
Avicel RC-581	Dupont	RC581-NFDR080I

### siRNA gene knockdown

siRNA and siRNA controls were ordered from ThermoFisher. Knockdowns were performed for 2 days using Lipofectamine RNAiMAX (Thermo Fisher Scientific) following the manufacturer’s instructions. See Table 2 for details on siRNA used.

### Quantitative reverse transcription polymerase chain reaction

Cells in 24-well plates were lysed in 200 µL Trizol at 0, 6, 12, 24, and 48 h post-infection. Four replicates were collected for each time point. Total RNA was extracted using the Direct-zol-96 RNA kit from Zymo Research. Five microliters of RNA (≤200 ng/µL) was incubated with random hexamer primers for 5 min at 70°C, then cDNA was synthesized using ImProm-II reverse transcriptase (Promega). To measure SARS-CoV-2 replication, quantitative real-time PCR was performed on 2 µL of cDNA product using AmpliTaq Gold DNA polymerase (Thermo Fisher) for 40 cycles with primers and probes specific for SARS-CoV-2 N: forward primer, 5′ GACCCCAAAATCAGCGAAAT-3′; reverse primer, 5′ TCTGGTTACTGCCAGTTGAATCTG 3′; probe, 5′ 6FAM-ACCCCGCATTACGTTTGGTGGACC-BHQ1 3′. RQ (2^−ΔΔ*Ct*^) values were calculated for each sample by normalizing to beta actin using specific primers and probes: forward primer, 5′ GGATCAGCAAGCAGGAGTAT 3′; reverse primer, 5′ AGAAAGGGTGTAACGCAACTAA 3′; probe, 5′ 6FAM-TCGTCCACC-ZEN-GCAAATGCTTCTAGG-IABkFQ 3′. The following samples had no detectable beta-actin amplification and were excluded from analysis: siCtrl 24 h, siUBE2D3 12 h and 48 h, siUBE2D3 + MYCBP2 24 h and 48 h. Data were plotted in GraphPad Prism and statistical significance was determined by ANOVA and Dunnett’s multiple comparisons test. See Table 3 for list of primers and Table 2 for details of enzymes used.

**TABLE 3:** Key resource table—primers

Primer target	Sequence 5' → 3'
SARS-CoV-2 N forward	GACCCCAAAATCAGCGAAAT
SARS-CoV-2 N reverse	TCTGGTTACTGCCAGTTGAATCTG
SARS-CoV-2 N probe	6FAM-ACCCCGCATTACGTTTGGTGGACC-BHQ1
Human beta actin forward	GGATCAGCAAGCAGGAGTAT
Human beta actin reverse	AGAAAGGGTGTAACGCAACTAA
Human beta actin probe	6FAM-TCGTCCACC-ZEN-GCAAATGCTTCTAGG-IABkFQ

### Structural mapping of residues

The RdRp complex consisting of NSP12, NSP7, and 2×NSP8 was downloaded from the Protein Database (PDB ID 9pyw), and UCSF ChimeraX (1.12rc202605220400 [22 May 2026]) was used to visualize and map residues of interest.

## SUPPLEMENTAL MATERIAL

Supplemental figures msphere.00373-26-s0001.docx Fig. S1 to S3.

ASM does not own the copyrights to Supplemental Material that may be linked to, or accessed through, an article. The authors have granted ASM a non-exclusive, world-wide license to publish the Supplemental Material files. Please contact the corresponding author directly for reuse.
