# Supplementary material for: Serine ubiquitination of the SARS-CoV-2 RdRp by the non-canonical E3 ligase MYCBP2
Source: mSphere. 2026 Sep 1;11(9):e00373-26. doi: 10.1128/msphere.00373-26 (PMC13621839; doi:10.1128/msphere.00373-26)
Supplement: Supplementary file 1 [file msphere.00373-26-s0001.docx]

 **Supplementary figure 1: A.** HEK293T cells were transfected with the indicated HA-tagged expression SARS-CoV-2 expression plasmid, followed by immunoprecipitation using anti-HA beads. IPs and WCLs were probed for ubiquitin and HA. Yellow fever virus (YFV) NS5 was used as a positive control for a ubiquitinated viral protein.

**Supplementary figure 2**: **A.** Schematic representation of the double-immunoprecipitation experiment to test covalent interaction of ubiquitin chains with NSP12. HEK293T cells were transfected with HA-Ub and FLAG-NSP12. Lysates were first incubated with anti-FLAG beads to immunoprecipitate total NSP12 (ubiquitinated and non-ubiquitinated). Half of the resulting IP samples were stripped from the FLAG beads and re-immunoprecipitated using anti-HA beads to IP only ubiquitinated NSP12. **B.** IP samples described in **A** were probed for HA (ubiquitin) and FLAG (NSP12). The top panel demonstrates that NSP12 is covalently associated with poly-ubiquitin chains, and the middle and bottom panels show that a minority of total NSP12 is ubiquitinated when overexpressed in HEK293T cells.

**Supplementary figure 3:** **A.** Multiple sequence alignment of the coronavirus NSP12s used in this study focusing on the region spanning S564, indicated by an arrow. **B.** Structure of NSP12 (blue) with NSP7 and NSP8 (grey) and RNA (pink). The red box in the left panel is magnified in the right panel, showing the locations of S564 (red) and T656 and T567 (orange).
